# Notices of the Influenza and Measles, as They Appeared at Cincinnati, in 1831 and 1832

**Published:** 1832

**Authors:** Daniel Drake


Art. IV.—*Notices of the Influenza and Measles, as they appeared at Cincinnati, in* 1831—2. By Daniel Drake, M. D.

Few epidemic diseases, are of more frequent occurrence, or exhibit more uniformity in their symptoms, than Influenza and Measles. To write the history of every invasion, would be tiresome and monotonous; but as these maladies are said by Webster, in his learned work on Epidemics, generally, to have preceded diseases of a pestilential character, and as Europe is now ravaged by Cholera, it may not be useless to record a notice of their late invasions.

The Influenza of 1831—2, commenced, with severity, not far from the middle of November, and the first paroxysm lasted for two months. The snows were of considerable depth, and the cold so intense and steady, as to bridge the Ohio with ice from its source to its mouth. At this place, horses and wagons crossed it for weeks.

The autumn of 1831 was not remarkable, either for the number, violence, or peculiarity of its cases of remittent and intermittent fever; and when, at the usual period, these ceased to occur, our city was free from any prevalent disease. It was in this state of the public health, that the Influenza suddenly appeared in the latter part of November, and afterwards prevailed for the ordinary time.

In its symptoms this epidemic presented nothing remarkable. In the majority of cases it was a simple coryza, or inflammatory irritation of the mucous membrane, of the nasal membrane and the sinuses opening upon it. But others presented all the signs of a bronchitis, more or less intense. When the disease proved fatal, it was with these symptoms. Adults were more liable to the malady, than children; and, in general, suffered more severely. In a few cases, it was attended with extreme prostratiod of strength [not to be accounted for by the pulmonary engorgement] apparently indicating the impress of an ærial poison on the nervous system. I saw this trait of pathological character, still more strikingly displayed, in the Influenza of 1825—6, when many patients, for a short time, had symptoms, not unlike those attendant on malignant congestive fever.

The appropriate treatment of the late Influenza, was the antiphlogistic. Blood letting was required in all the violent cases, especially when the greatest mucous irritation was in the bronchial tubes and cells, instead of the anterior ærial passages. I have reason to believe, that the inadequate employment of the lancet, was in some cases the cause of death—in a still greater number, of bronchial consumption.

But all cases did not require blood letting. In many, a general phlogistic action, was but little developed. The lungs were engorged, rather than inflamed. Such cases yielded readily to emetics—cathartics, with the subsequent use of sudorifics; a treatment which was attended with happy effects, in cases decidedly inflammatory, after copious venesection. Under this practice, a great quantity of bile was frequently discharged, with signal relief of all the symptoms. In violent cases, after blood letting, tarter emetic administered in grain doses, in the form of pills, alone or in combination with opium, and repeated every two hours, subdued the remains of inflammatory action, promoted expectoration and mitigated the dyspnoea, to which, I need scarcely add, a favorably convalescence immediately ensued.

I am somewhat surprized, that this mild form of the Rasorian practice, does not find greater favor with the physicians of America, in the treatment of acute bronchitis, pneumonitis, and other violent affections of the lungs. I have had much reason to be pleased with it, in my own practice; and have, in many cases, received from my medical brethren, satisfactory accounts of its tendency, when I have persuaded them to employ it. I look upon it, not exactly as a substitute for blood letting, but for *further* blood letting. In all violent cases, that evacuation should precede its employment. On the other hand, when the system has become irritable from depletion, while the pulmonary engorgement, is still such as to embarrass the respiration of the patient, it is proper to combine opium with the tartante.—Without an attention to these two points, the good effects of this practice can be but partially obtained.

As in endemic and sporadic catarrh, every case of Influenza did not require blood letting and other antiphlogistics, of an active character. Diluents, demulcents and sudorifics, and gentle narcotics, were successful in the milder cases, the number of which was not a few. These were attended with free expectoration, from the beginning; and, in their progress, frequently called forth the use of bark and a generous diet.

No invasion of Influenza, ever fails to awaken pulmonary consumption in the predisposed. The late epidemic has left behind it, less than the usual proportion of such cases, at least within the circle of my practice; but to what this mitigation may be ascribed, I am unable to say.

### MEASLES.

After two months of unabated frost, the latter part of January became mild. A thaw ensued with S. W. wind and copious rains. These were especially profuse on the streams which enter the Ohio and Alleghany from the north. Both rivers rose rapidly, and by the 18th of February, attained to an unprecedented height. A large proportion of their interval lands was overflown. Cold weather succeeded to the subsidence of the waters, and the months of March, April, and May, were generally dry, and much reduced in temperature; so that it may be safely affirmed, that during the six months ensuing from the middle of November 1831, there was more cold, at Cincinnati, than in any other six months since its settlement in 1788.

The prevailing disease in the latter part of the period here designated, was the Measles; which began in February, and has not yet (June 20,) entirely disappeared. It was most prevalent and fatal in April. At no previous time, has the malady excited so much alarm in this city. I could not discover, however, that it was more fatal than it has been in some other years.— The number of cases even in proportion to the increased population, was, it is true, greater, perhaps, than in previous attacks. I met with several well marked recurrences of the disease in those who had experienced it before. I, also, saw some adults affected, who had often been exposed to the infection in previous epidemics without contracting the malady. But such phenomena are not uncommon. Notwithstanding its prevalence for three months, as the sole epidemic, all the children of the city were far from being infected. I know several families, in which a part only have been attacked; and others, which escaped entirely; although the disease prevailed in their immediate neighborhood. In several, indeed a majority of cases, the malady could not be traced up to a communication with the sick, Several years since, on the other hand, it prevailed for many weeks in Cincinnati, without appearing in the village of Newport, on the opposite side of the Ohio; a fact that would seem to establish propagation by contagion. To this, however, the following case stands in direct opposition.—During the late epidemic, a child was taken from Cincinnati to a district of country, which was entirely free from the epidemic. Soon afterwards it sickened with the measles, and a number of children, were in constant intercourse with it, throughout the whole course of the disease; but not one of them was infected. Such a fact is not less embarrassing to the theory of contagion, than the exemption of the surrounding country, while Cincinnati was invaded, is to the theory of insensible, atmospheric meteoration.

In its symptomatology, the disease presented nothing remarkable. I met with two cases, which seemed to be the offspring of the morbillous poison, for they occurred in families afflicted with the malady, and commenced with the usual catarrhal symptoms, and yet differed so widely from the rest, that I shall notice them here. In one, the bowels were obstinately costive, but at length acted freely. Very considerable dyspnœa was present. The child was bled twice (after the period for the appearance of the eruption had passed) till syncope commenced; but the fever and difficulty of breathing returned. Antimonials, calomel, diluents and blistering were liberally employed. On the 8th day, a slight efflorescence appeared about the neck, and on the 9th the patient expired. Had this been a simple catarrhal affection, it must have yielded, to the copious bleedings; their inefficacy and the final appearance of a partial efflorescence, sufficiently indicate the impress of the remote cause of the epidemic.

The other case was attended with much less fever, as indicated either by the heat of the surface or the force of the pulse—both of which, indeed, were defective. Emetics, gentle narcotics, warm and stimulating diluents, the warm bath and blisters, were tried, industriously, but to no purpose. As in the other patient, a limited efflorescence appeared on the upper part of the trunk of the body about the 8th day, and in the following night the disease terminated fatally. The books speak of cases of *rubeola sine catarrho*,—these were examples of *rubeola sine efflorescentia*.

In most cases of the epidemic, the disease exhibited an open inflammatory character. The heat of the surface was considerable, and the fever ran high, especially in the first part of the night. The stomach was not more irritable, perhaps less so, than I had seen it in previous invasions of the same malady, nor were the lungs more deeply effected.

The proper treatment was the antiphlogistic, of which fresh and cool air was one of the most important items. I should think it unnecessary to make this statement, had I not seen many children injured by being kept under opposite circumstances; several by the advice or consent of their physicians, and more from the prejudices of the people. I am quite convinced, that during the two first days of the eruptive fever, indeed till near the time for the efflorescence, the cooler the patient is kept the better. When the eruption is about to shew itself, some reserve, in this respect, is necessary, and, occasionally the hot regimen is required, to throw a requisite quantity of blood into the skin. After the efflorescence has become established, a close and heated atmosphere is prejudicial; but great exposure on the other hand is injurious. In the decline of the cutaneous affection, and for several days afterwards, it is necessary to seclude the patient carefully from a cool and damp atmosphere; as chilliness at that time may produce fatal congestions of the viscera, particularly the lungs. Such are the precautions which I have found necessary in regard to heat and cold.

Bloodletting was frequently employed, and often with excellent effect. Every acute, or even violent case of measles, does not, however, call for venesection; nor will morbillous patients bear the lancet, like catarrhal, from changes of weather. Of this I have assured myself by repeated observations; having often seen a strong tendency to syncope under the loss of what, for the age of the patient, was a small quantity of blood. Altho' the stage of excitement be acute, and even violent, it does not therefore follow that bleeding should be employed. The disease has its *stadia* and *must* pass through them. The symptoms may be threatening, but, without reducing the powers of the system, they may in due time subside. When great dyspnœa, pain in the side, coma, convulsions, obstinate constipation, or excessive irritability of stomach, indicate the existence of a visceral, morbillous inflammation, so intense as to endanger the organ in which it is seated, then, and then only, would the lancet seem to be required.

Antimonials, preparations of squill, calomel, Dover's powder, demulcents, the tepid bath, and, in some cases, blistering were employed with advantage. It has appeared to me, however, on the whole, that too much was attempted by art, and too little left to nature—that is, the patient's instincts, which would lead him to moderate the eruptive fever by cool air, cold drinks, and abstinence—after which every thing would in general be well with him.

On the decline of the efflorescence, many patients were speedily well; but others continued feeble and anxious; experienced slight paroxysms of fever at night, with a dry and harsh skin, slept badly, and had more or less of diarrhœa, with tongues that were dry, brown, and cracked on the surface. Under these circumstances the tepid bath and small doses of magnesia in milk or mucilage, with a liberal use of lemonade, were of great service. I also found the following composition highly beneficial:—

R. Pulv. Gum Arab: two drachms,
Sacch: Albs: two drachms,
Ol. Ricine, four drachms,
Aq: Common Ferv: oz.iiss,

Triturate the three first together, and add the last gradually.

To this mixture, some aromatic oil, or a little laudanum, or paregoric was occasionally added.

An excellent, perhaps a better narcotic, in these cases, was the following:

R. Thick Mucilage of Gum Arabic,
Simple Syrup, each oz.i,
Sulphate of Morphia, gr. i,
Mix.

One case of the kind just mentioned, proved fatal; which, with the two cases of supposed measles above described, constituted the amount of my loss during the epidemic.

While the measles were prevailing in Cincinnati, without invading the country around, many parts of the latter were visited by a mild scarlet fever; which has not yet shown itself in the city.

June, 1832.

